# Role of Melatonin in the Management of Sleep and Circadian Disorders in the Context of Psychiatric Illness

**DOI:** 10.1007/s11920-022-01369-6

**Published:** 2022-10-13

**Authors:** Eunsoo Moon, Kyungwon Kim, Timo Partonen, Outi Linnaranta

**Affiliations:** 1 Department of Psychiatry, Pusan National University School of Medicine, Yangsan, Republic of Korea; 2 Department of Psychiatry and Biomedical Research Institute, Pusan National University Hospital, Busan, Republic of Korea; 3 Department of Public Health and Welfare, Finnish Institute for Health and Welfare (THL), P.O. Box 30, 00271 Helsinki, Finland

**Keywords:** Melatonin, Melatonergic agents, Sleep, Circadian rhythm, Circadian disorder, Psychiatric illness

## Abstract

**Purpose of Review:**

We present a review of research on the role of melatonin in the management of sleep and circadian disorders, stressing current overall view of the knowledge across psychiatric disorders.

**Recent Findings:**

Dysregulation of sleep and circadian rhythms has been established in several psychiatric and neurocognitive disorders for long. Recent research confirms this finding consistently across disorders. The secretion of melatonin in schizophrenia and neurocognitive disorders is reduced due to a smaller volume and enlarged calcification of the pineal gland. On the other hand, melatonin dysregulation in bipolar disorder may be more dynamic and caused by light-sensitive melatonin suppression and delayed melatonin secretion. In both cases, exogenous melatonin seems indicated to correct the dysfunction. However, a very limited number of well-designed trials with melatonin to correct sleep and circadian rhythms exist in psychiatric disorders, and the evidence for efficacy is robust only in autism, attention deficit hyperactivity disorder (ADHD), and neurocognitive disorders. This topic has mainly not been of interest for recent work and well-designed trials with objective circadian parameters are few. Overall, recent studies in psychiatric disorders reported that melatonin can be effective in improving sleep parameters such as sleep onset latency, sleep efficiency, and sleep quality. Recent meta-analysis suggests that optimal dosage and dosing time might be important to maximize the efficacy of melatonin. The knowledge base is sufficient to propose well-designed, larger trials with circadian parameters as inclusion and outcome criteria. Based on the partly fragmentary information, we propose testing efficacy in disorders with neurocognitive etiopathology with later and higher dosing, and affective and anxiety disorders with lower and earlier dosing of melatonin.

**Summary:**

Melatonin is promising for the correction of sleep and circadian abnormalities in psychiatric disorders. However, research results on its effect are still few and need to be accumulated. For effective use of melatonin, it is necessary to consider the appropriate dosage and administration time, depending on the individual abnormality of sleep and circadian rhythms.

## Introduction

The central role of circadian rhythms, representing biological oscillation around 24 h, is increasingly acknowledged in human health [1]. In the field of clinical psychiatry, abnormalities of sleep and circadian rhythms are more a rule than exception [2, 3•]. The abnormalities of circadian rhythms are related to negative outcomes such as symptom aggravation and recurrence in psychiatric disorders [4, 5]. Accumulating evidence suggests that correction of abnormal sleep and circadian rhythms beyond psychiatric symptoms to improve outcome in psychiatric disorders is warranted.

Melatonin is a key hormone regulating both sleep and circadian rhythms [6]. In psychiatric disorders, abnormalities of melatonin secretion and regulation have been reported [7, 8]. These might be due to either decreased secretion from the pineal gland, or dysregulation of the secretion due to internal or external reasons [9, 10].

Considering the commonly reported dysregulation of melatonin or other circadian rhythm abnormalities in psychiatric disorders, especially while symptomatic [3•], we need appropriate treatments to correct these abnormalities. A recent systematic review and meta-analysis reported positive effects of exogenous melatonin on sleep quality in adults with metabolic disorders, respiratory diseases, and primary sleep disorders, while no statistically significant findings were reported for individuals with mental disorders and neurodegenerative diseases [11]. However, population with mental disorders in the cohorts of this systematic review and meta-analysis was not representative of individuals of mental health disorders; rather, the cohorts included patients with an alcohol use disorder, schizophrenia, or bipolar disorder during benzodiazepine withdrawal [11]. Administration of exogenous melatonin has proven useful in the management of specific sleep and circadian disorders. The effects of exogenous melatonin on sleep and circadian rhythm are mainly mediated by melatonin receptor 1 (MT1) and 2 (MT2) [12–14]. MT1 is closely related to phase shifting of circadian rhythms and REM sleep regulation, and MT2 can be associated with NREM sleep regulation [12–14]. Whether the findings are relevant for treatment of all patients with psychiatric disorders remains open. In this article, we review pivotal and recent studies on effects of exogenous melatonin on sleep and circadian rhythms in psychiatric disorders. Based on the current accumulated evidence, we integrate the knowledge on the role of melatonin in the management of sleep and circadian disorders in the context of psychiatric illness.

## Role of Melatonin in Sleep and Circadian Rhythm Disorders

The effects of melatonin in cohorts with distinct sleep and circadian rhythm disorders are well established. In patients with primary insomnia, exogenous melatonin demonstrates significant effects on several sleep parameters such as sleep onset latency, total sleep time, morning alertness, and sleep quality, as reviewed in several recent systematic reviews and meta-analyses [15, 16]. A meta-analysis in primary insomnia with 5 original studies showed that exogenous melatonin significantly shortens sleep onset latency [15]. Most of the studies included in the Auld et al. meta-analysis administered melatonin 2 mg at 2 h before bedtime [15]. A recent randomized controlled trial (RCT) examined the effects of a higher dosage, 3 mg melatonin for 4 weeks, on sleep disturbance in 97 middle-aged patients with primary insomnia [17]. While this study showed a positive impact on sleep parameters, such as decreases in early wake time and percentage of N2 sleep, there was no effect of melatonin on a circadian marker, sleep onset latency [17].

Until now, only one, older RCT compared the efficacy on sleep onset latency according to melatonin dosage [18]. This study reported differential effects of melatonin as 0.1 mg, 0.3 mg, and 3 mg on several sleep parameters in 30 insomniac subjects who were over 50 years of age [18]. Although each melatonin administration did not show a difference in sleep onset latency compared to placebo, melatonin 0.3 mg showed the largest impact on sleep efficiency.

Further evidence for the potential of melatonin in correcting circadian rhythms is provided in some other target groups, including both disorders and external causes for a phase shift. A meta-analysis with a focus on patients with delayed sleep–wake phase disorder (DSWPD) concluded that exogenous melatonin advanced endogenous melatonin onset and shortened sleep onset latency [19]. Later, one RCT using 0.5 mg melatonin at 1 h before a desired bedtime significantly shortened sleep onset latency [20]. These studies propose usefulness of low-dosage melatonin administration in advancing sleep phase or shortening sleep latency. The improving effects of melatonin on sleep parameters have also been observed in subjects with jet lag symptoms [21, 22], in shift workers [23, 24], and in non-24-h sleep–wake rhythm disorder (N24SWD) [25, 26]. However, some older studies provide conflicting findings [21, 22, 24–26].

## Role of Melatonin on Sleep and Circadian Disorders in the Context of Psychiatric Illness

Abnormalities of sleep and circadian rhythms in psychiatric illness are summarized in Table 1. Furthermore, recent evidence on the effects of exogenous melatonin on sleep and circadian disorders was integrated in psychiatric disorders such as autism spectrum disorder, attention deficit hyperactivity disorder, neurocognitive disorder, schizophrenia, bipolar disorder, depression, anxiety, and eating disorder (Table 2).

**Table 1:** Abnormalities of sleep and circadian rhythms in psychiatric illness

**Study population**	**Principal abnormalities of sleep and circadian rhythms in psychiatric illness**
Autism spectrum disorder	Mutations in circadian clock regulating genes [30] Longer sleep onset latency, lower sleep efficiency, decreased total sleep time, and lower amplitude [29]
Attention deficit hyperactivity disorder	Delayed sleep–wake phase disorder [39] Higher or delayed melatonin levels [28]
Neurocognitive disorder	Decreased pineal gland volume, pineal calcification [9, 41]
Schizophrenia	Circadian misalignment, irregular and fragmented sleep [46] Decreased pineal gland volume [44] Longer total sleep time, longer time in bed, greater sleep latency, increased wake after sleep onset [2]
Bipolar disorder	Delayed phase of melatonin and cortisol, Eveningness chronotype [50] Light-sensitive melatonin suppression and delayed melatonin secretion [10] Longer total sleep time, longer time in bed, greater sleep latency, increased wake after sleep onset, decreased sleep efficiency [2]
Depression	Delayed melatonin onset, advanced sleep onset, increased total sleep time [102] Eveningness chronotype [60]
Anxiety	Positive association with melatonin level after lunch [68]
Eating disorder	Melatonin dysregulation [72] Decreased midline estimating statistic of rhythm (MESOR) and amplitude of rest-activity circadian rhythm [73] Robust association with irregular eating pattern and late sleep phase [103]

**Table 2:** Effects of exogenous melatonin on sleep and circadian disorders in the context of psychiatric illness

**Study population**	**Evidence of melatonin on sleep and circadian disorders in the context of psychiatric illness**
Autism spectrum disorder	Shortened sleep latency, increased total sleep duration (0.5 ~ 12 mg) [28]
Attention deficit hyperactivity disorder	Shortened sleep latency, increased total sleep duration (3 ~ 10 mg) [28] Advanced dim-light melatonin onset (DLMO) (0.5 mg) [40]
Neurocognitive disorder	Enhanced rest-activity rhythm, improved sleep quality, increased morning alertness, decreased sleep onset latency (1 ~ 24 mg) [42•]
Schizophrenia	First-night effects (2 mg) [48] Improved sleep efficiency (2 mg) [47]
Bipolar disorder	No randomized controlled trial
Depression	Improved sleep quality (5 ~ 10 mg) [63]
Anxiety	Improved sleep quality, sleep duration, sleep latency, sleep efficiency (6 mg) [71]
Eating disorder	No randomized controlled trial

### Autism

Several studies reported abnormalities of sleep and circadian rhythms in patients with autism, as reviewed recently [27–29]. Based on these reviews, patients with autism showed longer sleep onset latencies, lower sleep efficiencies, decreased total sleep times, and lower amplitudes [29]. Mutations in circadian clock-controlled genes were also found in patients with autistic spectrum disorder [30]. Additionally, the melatonin levels in autism were lower than in healthy controls [28, 30]. The circadian dysfunction in patients with autism might be related to the pathogenesis of neurodevelopmental disorders such as autism [31•]. Especially, abnormalities in the sense of time, which are hypothesized as “temporal binding deficit” or “social timing hypothesis,” were observed in high-functioning autism patients as well as in severely symptomatic patients [31•]. Furthermore, disruption of circadian rhythms might affect immune-inflammatory, oxidative, and metabolic pathways, as well as neurotransmission underlying the biological mechanisms of autism [8].

Several studies indicated the efficacy of exogenous melatonin in autism spectrum disorders. One review on 15 original trials summarized that exogenous melatonin in autism spectrum disorders shortened sleep latencies and increased total sleep durations, as reviewed recently [28]. Based on the evidence, a clinical guideline for autism [32] recommended melatonin 2 mg as an initial dose and increased up to 5 mg depending on the severity of sleep problems, as opposed to low dosages less than 1 mg used for chronobiotic effects. Higher dosages of melatonin in autism spectrum disorders would be reasonable for improving disrupted melatonin rhythms rather than low-dose melatonin administration [32–34], considering the findings on dysfunction of circadian rhythms and attenuation of melatonin rhythm.

### ADHD

Sleep and circadian rhythm abnormalities in patients with attention deficit hyperactivity disorder (ADHD) have frequently been reported, as reviewed by Martinez-Cayuelas et al. and Bondopadhyay et al. [35, 36]. Daytime and nocturnal activities were increased, and the post-lunch dip in alertness was absent [37, 38]. Melatonin levels in ADHD were higher or delayed as compared to those in healthy controls, as reviewed recently [28]. The delayed sleep–wake phase disorder was frequently reported in patients with ADHD [39].

A recent systematic review summarized findings from 5 RCTs in ADHD and concluded that exogenous melatonin administration can shorten sleep latencies and increase total sleep durations in ADHD [28]. Most of the studies included in the Rzepka-Migut and Paprocka review used a relatively high dosage, ranging from 3 to 10 mg. Again, considering that the delay in melatonin rhythm may be a target problem in ADHD, it seems necessary to advance the circadian rhythm with a low dose of melatonin. A recent RCT examined the effects of melatonin monotherapy or melatonin with bright light therapy in adult ADHD patients with delayed sleep phase syndrome [40]. In this study, 0.5 mg melatonin for 3 weeks significantly advanced the dim-light melatonin onset (DLMO) compared to placebo [40]. This study also reported that melatonin supplements may be more useful when used in conjunction with bright light therapy.

### Neurocognitive Disorders

Abundant data shows that neurocognitive disorders such as dementia or mild cognitive impairment are commonly accompanied by abnormalities of sleep and circadian rhythms. The abnormalities might be related to decreases in the pineal gland volumes or pineal calcification [9, 41]. Given that the pineal gland dysfunction implies the necessity of melatonin supplement, using melatonin for part of treatments of neurodegenerative disorders is warranted and supported by several trials.

One comprehensive review on 14 original studies reported that melatonin supplements in Alzheimer’s disease improved sundowning, sleep quality, and rest-activity disturbances [42•]. In this review, most clinical studies in Alzheimer’s disease used relatively high dosages of melatonin up to 10 mg, mostly administrated at bedtime [42•]. In addition, melatonin supplements with a wide range of dosage from 1 to 24 mg in mild cognitive impairment enhanced rest-activity rhythms and improved sleep profiles such as sleep quality, morning alertness, and sleep onset latency in a comprehensive review including 11 studies [42•]. Melatonin can be a useful treatment for correcting sleep and circadian rhythm abnormalities in neurodegenerative diseases. When considering the aspect of compensating the abnormal melatonin secretion rather than the advancing effect of melatonin rhythm, it might be reasonable to opt for high doses of melatonin. Furthermore, high doses of melatonin have potential antioxidant effects, with speculative benefits in neurodegenerative diseases [42•, 43].

### Schizophrenia

Abnormalities of melatonin secretion in schizophrenia have been consistently reported in many studies. The calcification of the pineal gland in patients with schizophrenia has frequently been observed [44]. Recent meta-analyses reported a reduced volume of the pineal gland, and reduced midnight plasma levels of melatonin in schizophrenia [44, 45]. Also, these abnormalities of the melatonergic system might be plausibly connected to the occurrence of sleep and circadian rhythm abnormalities, such as sleep disturbances, delayed/advanced circadian misalignment, or non-24-h irregular sleep–wake patterns in schizophrenia [2, 46].

Given the abnormalities of the melatonergic system in schizophrenia, the correction of sleep and circadian rhythms using melatonin administration in treatment of patients with schizophrenia might be a clinically meaningful approach, but the evidence for efficacy remains preliminary, and there are no recent studies. Two small randomized controlled trials (RCTs) examined the efficacy of exogenous melatonin in schizophrenia [47, 48]. One RCT with 19 schizophrenia patients reported that controlled-release melatonin 2 mg significantly improved sleep efficiency compared to placebo [47]. Meanwhile, another RCT in 14 patients with schizophrenia examined the first-night effect that represents sleep disturbances under the unfamiliar sleeping environment using polysomnography [48]. This study reported that controlled-release melatonin 2 mg increased rapid eye movement (REM) sleep latency, decreased sleep efficiency, and increased wakefulness after sleep onset (WASO) compared to placebo. A recent systematic review synthesized the clinical evidence on therapeutic use of melatonin in schizophrenia [49]. Beyond sleep problems, melatonin supplements have been tried in various clinical situations in patients with schizophrenia such as alleviation of antipsychotic-induced metabolic adverse effects, alleviation of tardive dyskinesia, amelioration of cognitive dysfunction, and discontinuation or reduction of benzodiazepines. This systematic review reported the positive effects of adjunctive melatonin on sleep, metabolic profile, and tardive dyskinesia in patients with schizophrenia, not on cognition or benzodiazepine discontinuation [49]. During the past over 10 years, no RCT with melatonin on sleep or circadian abnormalities in schizophrenia has been conducted.

### Bipolar Disorders

Strong evidence shows that patients with bipolar disorders commonly show sleep and circadian rhythm abnormalities, even while euthymic. The characteristics include irregular sleep–wake rhythm, sleep fragmentation, decreased nocturnal melatonin levels, increased light-induced melatonin suppression, delayed phase of the dim-light melatonin onset (DLMO), and abnormal melatonin synthesis, as reviewed by several authors [10, 50–53]. Recent work adds variations in sleep midpoint and decreased sleep consolidation to this evidence [54]. An association between sleep and eating rhythms as well as a complex relationship of sleep with current mood status provides support for an internal rhythmopathy in bipolar disorders [54]. Longitudinal studies are necessary to understand the individual-level dynamics of sleep phasing. While information was found only from one study with 31 mood episodes of 26 patients with bipolar disorder, and 18 controls, it seems that while a depressive phase is associated with a phase delay, a manic phase is associated with a phase advance [53].

The patterns of sleep and circadian rhythm abnormalities may be different in bipolar disorder from those in schizophrenia. The secretion of melatonin in schizophrenia is reduced due to a smaller volume and enlarged calcification of the pineal gland [44]. On the other hand, melatonin dysregulation in bipolar disorder may be caused by light-sensitive melatonin suppression and delayed melatonin secretion [10]. A recent systematic review and meta-analysis compared sleep and circadian parameters in bipolar disorders and schizophrenia [2]. The parameters of sleep disturbances, such as sleep onset latency (SOL), wake after sleep onset (WASO), and total sleep time (TST), indicated smaller abnormalities in bipolar disorders than in schizophrenia [2]. However, comparative, longitudinal studies including a full range of circadian parameters are necessary to truly understand the dynamics of circadian regulation in these two diagnostic groups.

Despite the definite clinical need for evidence-based treatments to correct problems with sleep and circadian rhythms, no well-designed studies on the efficacy of melatonin administration in sleep and circadian rhythms in bipolar disorder were identified [55]. One RCT with two parallel groups, including a mixed mood disorder cohort of 33 patients with bipolar disorder or major depressive disorders, reported no significant effects of slow-release melatonin 6 mg on sleep parameters compared to placebo [56]. This study used a high dosage of melatonin, 6 mg at bedtime for 4 weeks. This does not exclude potential effect of a smaller dose with an earlier timing that was identified as the most potential to correct a delayed rhythm in our systematic review in other populations [84••].

### Depressive Disorders

Abnormalities in sleep and circadian rhythms are also reported in depressive disorders [57]. Patients with major depressive disorder showed decreased amplitudes and delayed/advanced phases of the circadian rhythms such as core body temperature and melatonin [58–60]. In patients with seasonal affective disorder, sleep disturbances and phase delays or advances of the circadian rhythms have been observed [61, 62].

Data on the efficacy of exogenous melatonin in major depressive disorder is too limited for conclusions, and no recent trials were identified. One old RCT comparing exogenous melatonin to placebo showed improvement in sleep quality [63]. Meanwhile, another RCT with a mixed sample of patients with major depressive disorder or bipolar disorder did not show significant effects of slow-release melatonin 6 mg on sleep parameters [56]. A systematic review and meta-analysis reported the therapeutic or prophylactic efficacy of melatonin supplements against depression [64]. The meta-analyses did not show any significant finding, but some studies implied potential efficacy of exogenous melatonin on depression [64].

### Anxiety and Anxiety Disorders

Stress and stress hormones at certain times of the day can shift peripheral oscillators, such as those in the liver, kidney, and heart [65]. Shifts of peripheral oscillators can change the circadian organization for adaptation to repeated stress exposures [65]. Stressful life events can disrupt circadian rhythms including cortisol rhythm [65, 66]. Anxiety-related traits, such as anxiety sensitivity, neuroticism, and perfectionism, are associated with disruptions of sleep and circadian rhythms [67]. Having anxiety disorder was associated directly with the melatonin levels after lunch [68]. The findings correspond to the clinical knowledge and indicate that having stress and anxiety can have an impact on rhythms regulating hormones and commonly disrupt sleep and circadian rhythms.

In heterogeneous clinical conditions, several recent trials have shown the potential of exogenous melatonin in the management of anxiety-related symptoms in somatic conditions with disturbances in sleep and circadian rhythms. In patients undergoing intravenous regional anesthesia, melatonin is associated with reduced anxiety, decreased tourniquet-related pain, and improved perioperative analgesia [69]. A Cochrane review concluded that melatonin reduced pre-operative and post-operative anxiety with an effect equal to benzodiazepines [70]. A recent RCT in colorectal cancer patients undergoing chemotherapy and having sleep problems showed that melatonin had significant effects on sleep quality at week 4 [71]. Also, the effects of melatonin and zolpidem on sleep duration, sleep latency, sleep efficiency, and sleep disturbance were similar [71]. However, we are not aware of any trials in cohorts with anxiety disorders or stress in specific.

### Eating Disorders

A limited number of studies have investigated patients with eating disorders. However, all of them report sleep and circadian rhythm abnormalities [72–74]. In the first study, patients with night-eating syndrome (*n* = 15) showed melatonin dysregulation and abnormalities in food intake and in leptin and insulin levels [72]. In female patients with anorexia or bulimia (*n* = 23), sleep problems were associated with eating disorders [75]. Also, compared with obese women without binge eating disorder (*n* = 8), obese patients with binge eating disorder (*n* = 8) showed decreased midlines estimating statistic of rhythm (MESOR) and amplitudes of the rest-activity circadian rhythm, whereas no difference in acrophases was detected [73]. A recent study with a mixed sample of patients with eating disorders (*n* = 29) reported that a late sleep phase was robustly associated with an irregular eating pattern, proposing shared dysregulation [74]. Taken together, several small studies show that dysregulated eating behaviors are associated with dysregulated circadian rhythms. However, we could not identify any study observing the therapeutic usage of melatonin in eating disorders. One clinical study was tried but terminated due to difficulties in recruiting participants [76].

## Adverse Effects of Melatonin

Melatonin is generally regarded as safe and well-tolerated, notably, as compared to other treatment options, especially benzodiazepines or other hypnotics [33, 77, 78]. A recent systematic review [79] identified 37 RCTs in which the most frequently reported adverse events were daytime sleepiness (1.66%), headache (0.74%), other sleep-related adverse events (0.74%), dizziness (0.74%), and hypothermia (0.62%). Very few adverse events being serious or of clinical significance were reported. These included agitation, fatigue, mood swings, nightmares, skin irritation, and palpitations. Most adverse events either resolved spontaneously within a few days with no adjustment for the dosage of melatonin, or immediately after the withdrawal. However, there are very few studies that specifically examine the safety of melatonin use in those aged 65 years or over [80].

Based on limited evidence, even a large dose of melatonin appears to have a good safety profile. A recent systematic review [81] on RCTs investigating high-dose melatonin (≥ 10 mg) in adults over 30 years of age was included. In total, 79 RCTs were identified with a total of 3861 participants. Overall, only four studies met the pre-specified low risk of bias criteria for meta-analysis. In that small subset, melatonin did not cause a detectable increase in severe adverse events (rate ratio = 0.88 [0.52, 1.50]) or withdrawals due to adverse events (0.93 [0.24, 3.56]), but it did appear to increase the risk of adverse events such as drowsiness, headache, and dizziness (1.40 [1.15, 1.69]). Based on the long-term clinical studies in children, exogenous melatonin was safe and did not associate with delays in growth or puberty, or tolerance [33, 78]. We were unable to identify RCT reports on the prevalence of adverse effects in psychiatric disorders.

## Clinical Consideration of Melatonin Usage in the Context of Psychiatric Illness

Sleep and circadian rhythm dysfunctions have been reported in all psychiatric disorders, but specific parameters which point them out differ from disorder to disorder. Knowledge on sleep and circadian parameters in some disorders such as bipolar disorders and neurocognitive disorders is robust, and more fragmentary in others, such as eating disorders. Knowledge on the individual-level dynamic changes in specific parameters over time and in relation to current symptom profiles is very limited.

The abnormalities of sleep and circadian rhythms are an essential therapeutic target for the treatment of psychiatric disorders. Commonly used psychoactive agents are not sufficient as such to correct but commonly even worsen problems with circadian rhythm [82, 83]. We consider melatonin and melatonergic compounds to be the most promising existing complementary therapeutic compounds to correct abnormalities of sleep and circadian rhythms. However, the number of well-designed trials to prove the efficacy of melatonin supplementation for the correction of sleep and rhythm dysregulation in psychiatric disorders is too limited to consider melatonin as an evidence-based treatment option. As summarized in this review, fragmentary data provides sufficient support for our proposal that the complementary use of exogenous melatonin in psychiatric disorders warrants well-designed future trials.

**Fig. 1:**
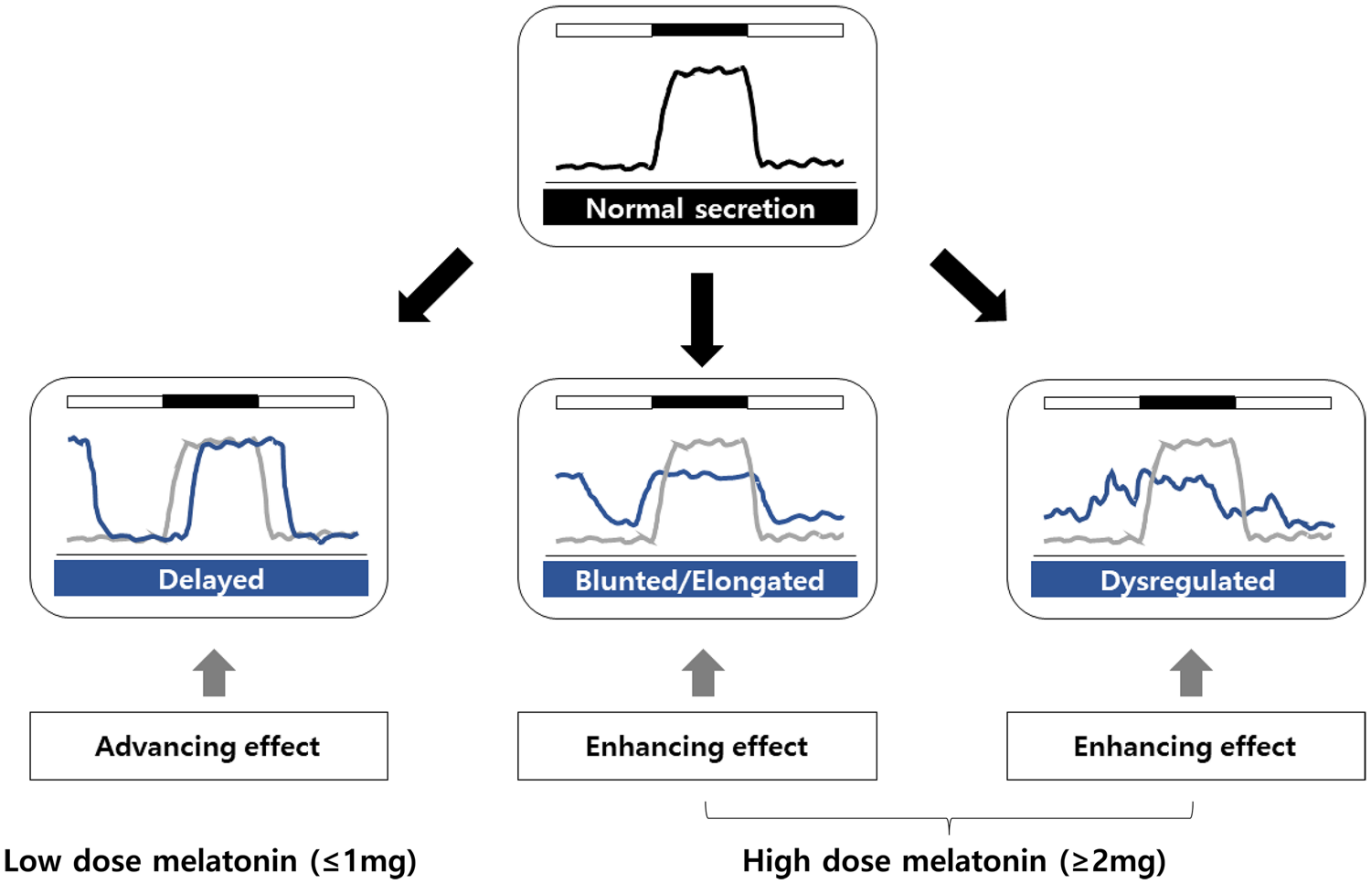
Potential types of melatonin dysfunction and target effects of exogenous melatonin supplement according to dosage. In case of delayed melatonin rhythm, low-dose melatonin (≤ 1 mg) with earlier timing is needed to advance the melatonin rhythm for a chronobiotic effect. Meanwhile, in cases of blunted or dysregulated melatonin rhythms, high-dose melatonin usage (≥ 2 mg) with later timing would be better to enhance the melatonin rhythm and improve sleep disturbance for a soporific effect

To maximize the efficacy of melatonin, it is necessary to check whether the dosage and dosing time of melatonin are used properly according to abnormalities of sleep and circadian rhythms. Our recent meta-analysis study showed that low-dose melatonin (≤ 1 mg) may be more effective at initiating sleep [84••]. Low-dosage melatonin such as 0.3 mg and 1 mg showed significant effects on shortening SOL, while relatively high-dosage melatonin such as 2 mg and 5 mg did not show a significant effect on SOL [84••]. Meanwhile, high-dosage melatonin 5 mg significantly increased sleep efficiency [84••]. As shown in Fig. 1, low-dose melatonin can sufficiently advance the phase of sleep and circadian rhythms as a chronobiotic agent [84••, 85••]. High-dose melatonin can efficiently show the soporific effects such as improving sleep efficiency and increasing total sleep time [84••, 85••].

In addition, administration time may be an important factor in advancing the circadian rhythm of melatonin. It may vary depending on the individual’s circadian rhythm, but according to the results of a meta-analysis, administration at 18:00 or 20:00 h may be the most effective [84••]. The administration time would be optimal around 3 h before the dim-light melatonin onset (DLMO) based on the phase-response curve [84••]. Therefore, in order to more effectively correct sleep or circadian abnormalities in psychiatric disorders, it is necessary to apply both an appropriate dose and an appropriate dosing time, according to the purpose of melatonin supplements [84••, 85••]. In previous studies, a low-dose melatonin (< 1 mg) could be effective for advancing sleep and circadian rhythms as a chronobiotic [86, 87]. The phase-response curve was different depending on the dose [86–89]. At doses as low as 1 mg, 2 h before DLMO may be the most effective dosing time to advance the rhythm [89]. On the other hand, at high doses such as 3 mg, the most effective dosing time may be 5 h before DLMO [89]. Given that the phase-response curve differs by the dosage, high doses of melatonin may not be beneficial to advance the melatonin rhythm.

The dosage and dosing time of melatonin could be different depending on the abnormalities of sleep and circadian rhythms as observed in patients with psychiatric disorders. We recently reviewed the objective methods of measuring sleep and circadian rhythms [90]. Clinically, actigraphy is the most suitable and useful objective device for measurement. The assessment of personal abnormalities in sleep and circadian rhythm could optimize the efficacy of melatonin supplements both clinically and in trials. There is lack of evidence on the proper use of melatonin according to the individual rhythms. However, if these results were to accumulate, it is expected that the effects of melatonin can be improved through personalized intervention according to each individual’s melatonin rhythm.

## Complementary Use of Melatonin and Other Indications for Use of Melatonin

Melatonin can be used because of its antioxidant and anti-inflammatory properties as well [91, 92]. With antioxidant properties on nigrostriatal dopaminergic system [93], melatonin supplementation was aimed to reduce tardive dyskinesia in patients with schizophrenia being treated with antipsychotics [94]. A meta-analysis suggested a potential efficacy for improving tardive dyskinesia, although no significant effect was demonstrated [94]. Antioxidant properties of melatonin might be utilized, e.g., to modulate mitochondrial dysfunction in aging, cognition, and psychiatric disorders such as schizophrenia and bipolar disorder [95]. Furthermore, melatonin might be a useful alternative as an anti-inflammatory agent [92, 96]. Dysfunction of immune system might be related to psychiatric disorders such as schizophrenia and mood disorders [97, 98]. Furthermore, melatonin may be beneficial for metabolic syndrome in patients treated with antipsychotics [99]. A recent systematic review and meta-analysis demonstrated the effects of melatonin on attenuating metabolic parameters such as fasting glucose, blood pressure, high-density lipoprotein, and triglycerides [99]. These effects might be direct or due to correcting sleep or circadian rhythms. Also, melatonin has been tried to treat insomnia caused due to benzodiazepine withdrawal. Currently, there are conflicting results. One RCT did not find significant effects of melatonin on the average benzodiazepine dosage, benzodiazepine cessation proportion, or withdrawal symptoms at 24 weeks [100]. However, melatonin supplementation in another RCT significantly improved the self-reported sleep quality [101]. Further studies are warranted to clarify the facilitating efficacy of melatonin on benzodiazepine withdrawal.

## Limitations

This article is not based on a systematic review, but here, we complemented our recent systematic review on melatonin supplementation [84••] with a comprehensive review that included recent research findings, a clinician-researcher view, and evidence for melatonin dysregulation in specific psychiatric disorders. Also, we did not include the studies on melatonergic agonists, such as ramelteon, tasimelteon, and agomelatine, but focused on the efficacy and safety of exogenous melatonin supplementation. This was done on purpose, given that the previous systematic review demonstrated that on this topic, there were a very limited number of trials in psychiatric disorders [84••].

## Conclusions

Abnormalities in sleep and circadian rhythms observed in psychiatric disorders can negatively affect the course of the disease. Therefore, clinical interest in sleep and circadian rhythm abnormalities and efforts to correct them are required. Several recent studies suggest that melatonin is a safe and well-tolerated treatment option to correct sleep and circadian rhythm disturbances, but studies evaluating the efficacy of melatonin as a complementary treatment in psychiatric disorders are too limited for conclusion. In order to maximize the effects of exogenous melatonin in sleep and circadian disorders, it is necessary to optimize the appropriate dose and administration time according to the type of circadian rhythm abnormalities. Future research trials should characterize the eligibility criteria and the assessment of outcome to be based on specific, objective parameters for circadian rhythms.
